# Preliminary Study of Ge-DLC Nanocomposite Biomaterials Prepared by Laser Codeposition

**DOI:** 10.3390/nano9030451

**Published:** 2019-03-18

**Authors:** Miroslav Jelinek, Tomáš Kocourek, Karel Jurek, Michal Jelinek, Barbora Smolková, Mariia Uzhytchak, Oleg Lunov

**Affiliations:** 1 Institute of Physics of the Czech Academy of Sciences, Na Slovance 2, 182 21 Prague 8, Czech Republic; kocourek@fzu.cz (T.K.); jurek@fzu.cz (K.J.); smolkova@fzu.cz (B.S.); uzhytchak@fzu.cz (M.U.); lunov@fzu.cz (O.L.); 2 Faculty of Biomedical Engineering, Czech Technical University in Prague, nam. Sitna 3105, 27 201 Kladno, Czech Republic; 3 Faculty of Nuclear Science and Physical Engineering, Czech Technical University in Prague, Brehova 7, 115 19 Prague 1, Czech Republic; michal.jelinek@fjfi.cvut.cz

**Keywords:** germanium, DLC, doped biomaterials, pulsed laser deposition, reactive oxygen species, apoptosis, cytotoxicity

## Abstract

This paper deals with the synthesis and study of the properties of germanium-doped diamond-like carbon (DLC) films. For deposition of doped DLC films, hybrid laser technology was used. Using two deposition lasers, it was possible to arrange the dopant concentrations by varying the laser repetition rate. Doped films of Ge concentrations from 0 at.% to 12 at.% were prepared on Si (100) and fused silica (FS) substrates at room temperature. Film properties, such as growth rate, roughness, scanning electron microscopy (SEM) morphology, wavelength dependent X-ray spectroscopy (WDS) composition, VIS-near infrared (IR) transmittance, and biological properties (cytotoxicity, effects on cellular morphology, and ability to produce reactive oxygen species (ROS)) were studied in relation to codeposition conditions and dopant concentrations. The analysis showed that Ge-DLC films exhibit cytotoxicity for higher Ge doping.

## 1. Introduction

Recent advances in the application of body implantable medical devices have created a demand for studies developing new robust biocompatible materials with improved physicochemical properties [1]. Materials that are biocompatible and/or have biocompatible end degradation products are critically important for such devices. Diamond-like carbon (DLC) films represent a promising material for the modification of medical implants, providing high mechanical and chemical stability and a high degree of biocompatibility [2,3,4].

DLC chemical modifications with silver, germanium, and copper have been utilized to further improve the surface properties of DLC coatings in terms of biocompatibility and optical, electronic, and mechanical properties [4,5,6,7,8]. Among the modifications, germanium (Ge)-based DLCs and alloys have received growing interest in studies relating to biomedical application due to their improved physicochemical properties [9,10,11,12,13]. For instance, a recent study showed that Ge-DLC films had better mechanical properties (high hardness, high durability, low stress, low absorption, broad band infrared (IR) transparency, variable refraction index from 1.7 to 4) and good adhesion to many IR substrates compared with pure DLC films [14]. Furthermore, germanium carbide (GeC) thin films were shown to be easily fabricated by pulsed laser deposition (PLD) at room temperature [15]. Another study revealed that, for concentrations below about 10 at.%, carbon atoms were incorporated in the germanium lattice and created stabile GeC alloy [16]. Moreover, changes to band gap were observed in region from 1.6 to 2.8 eV for GeC films [16]. Radio frequency plasma-enhanced chemical vapor deposition (PECVD) was shown to be effective in creating protective antireflective DLC coating on germanium [17]. Indeed, enhancement in maximum transmission above 90% in the 3–6 µm wavelength range was found [17]. Additionally, one can generate multilayer DLC and germanium-doped DLC films [18]. Such films possess hardness above 48.1 GPa, which is almost the same as that of pure DLC film [18]. Compared with the pure DLC film, the critical load of Ge-DLC film on the germanium substrate increases from 71.6 mN to 143.8 mN [18]. Moreover, Ge-DLC film showed no change after fastness tests [18]. Importantly, germanium-doped DLC films with different germanium concentrations showed similar properties [19]. Furthermore, germanium-carbon (GexC1-x) alloy coatings have been shown to have low infrared optical absorption [20]. The Ge-DLC films prepared by pulsed vacuum arc deposition and electron beam evaporation showed that the Ge-doping significantly improved the optical and mechanical properties of the films [21]. Hollow-cathode plasma-enhanced chemical vapor deposition was shown to be effective in generation of DLC and Ge-DLC coatings [22]. Such coatings showed a significant anti-biofouling effect on *Pseudomonas aeruginosa* [22]. In contrast, neither modified DLC nor Ge-DLC showed any significant inhibitory effect on *Staphylococcus aureus* [22]. The overall surface energy of the Ge-DLC coating was approximately 40% larger than that of undoped modified DLC [22]. The resultant wettability was also higher, and the polar component of surface energy for Ge-DLC was significantly larger [22].

From the above literature review, we can conclude that Ge-DLC films represent a promising material for a wide range of applications, including in optics and biomedicine. However, from a material point of view, the results and data presented to date have sometimes been contradictory and neither fully nor systematically related to deposition parameters and Ge concentrations. No study of the properties on a larger and finer scale of Ge dopant concentrations has been performed. Indeed, there is a potential applicability of Ge alloys as biocompatible thin films due to their improved physicochemical properties [10,11,12]. Specifically, studies of biocompatibility testing of Ge alloys have shown that such alloys have presumably low cytotoxicity profile and are well tolerated by the organism [10,11,12]. Nonetheless, from a biological point of view, the number of such studies is limited. Indeed, only a few works have addressed the long-term cytotoxicity of germanium-based alloys so far [10,11,12]. However, it is difficult to make any reasonable conclusion out of these papers, because they lack crucial data on positive and negative controls during toxicity assessment [10,11,12]. It is worth noting here that long-term administration of high-dose germanium products presents a potential human health hazard [23]. Thus, it is of great importance to study the cytotoxic effects of Ge alloys on sensitive biological systems.

This study aims to present and discuss preliminary results obtained on Ge-doped DLC layers. We conducted a physical and biocompatibility study of Ge-doped DLC layers directly connected with dopant concentrations. Furthermore, we investigated the in vitro adhesion, proliferation, and toxicity of one of the very sensitive cell lines (hepatic, Huh7) upon culturing on Ge-doped DLC layers using various bioassays. We combined physicochemical analysis (the growth rate, roughness, morphology, composition, transmission) of Ge-doped DLC layers with their biological properties (cytotoxicity, effects on cellular morphology, and ability to produce reactive oxygen species (ROS)) for Ge concentrations ranging from zero up to 12 at.%. Here, we show a preliminary study of the cytotoxicity of Ge-based substrates with a wide range of germanium concentrations. However, long-term acute effects of such substrates on physiological functions of the cells have not been thoroughly investigated. Additionally, with our study, we aim to show that Ge-based films might have significant adverse effects and might not be as safe as they are considered.

## 2. Experimental

**Figure 1:**
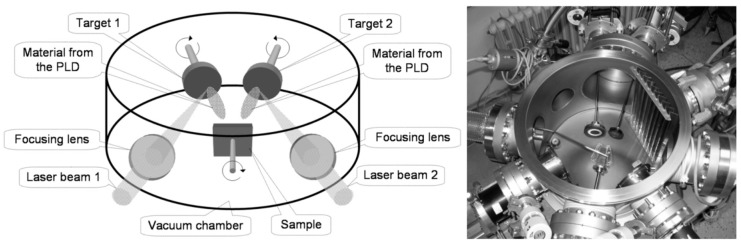
Dual beam laser deposition (schema—left, photo of chamber—right).

***Deposition***: Ge-DLC films were deposited by dual PLD using two krypton fluoride (KrF) excimer lasers (λ = 248 nm, τ = 20 ns), as shown in Figure 1. The flows of two target material were directed to rotating substrate. Our arrangement allowed, in a simple way, to prepare doped layers with a large scale of dopant concentrations by changing the laser fluence on targets and lasers’ repetition rates. The first laser Compex Pro (Coherent Inc., Santa Clara, CA, USA) beam was focused onto a high-purity graphite target with the energy density of 8 J cm^−2^. The repetition rate was set in the range from 18 Hz to 26 Hz. The second laser Lumonics PM-800 (Coherent Inc., Santa Clara, CA, USA) was focused onto a Ge target with the energy density of 1.5 J cm^−2^ and the repetition rate of 1 Hz to 23 Hz. The number of pulses was adjusted to reach approximately the same layer thickness. The substrate was in a distance of 40 mm away from the targets. The targets were rotated (0.5 Hz). To increase the films’ adhesion, the substrates were cleaned by radiofrequency (RF) discharge before deposition for 2 min at 100 W power. The base vacuum of the coating system was 5 × 10^−4^ Pa. The films were codeposited in argon ambient (0.25 Pa) at room substrate temperature. Substrates of Si (100) were used for study of morphology and composition, and FS substrates were used for biotesting. Deposition conditions are summarized in Table 1.

***Surface, thickness, roughness***: The surface of the samples was analyzed by mechanical profilometer Alphastep IQ—KLA Tencor (KLA Corporation, Milpitas, CA, USA) and by SEM. The profilometer scan was 2 mm long and 50 µm s^−1^ fast; sampling frequency was 50 Hz, sensor range 13 µm, and stylus force was 4.5 mg. A cut-off value of 250 µm was used for calculation of the roughness parameters. A stylus with a 5 µm tip radius and 60° angle was used. Two scans were performed in two perpendicular directions. The roughness average (Ra for line) was adapted from ISO 4287/1 and calculated by either Alphastep or Gwyddion software.

***Morphology, composition (SEM, WDS)***: The SEM morphology and composition of Ge-DLC thin layers was determined using an electron microprobe JEOL JXA 733 (JEOL Inc., Peabody, MA, USA) employing a wavelength dispersive X-ray spectrometer (WDS). The energy of primary electrons was kept at 3 keV to minimize their penetration depth and the absorption of emitted X-rays. For this energy, an electron spot diameter was estimated to be in the range 1–2 µm with the information depth of about 0.2 µm so that the contribution of the substrate could be neglected. In this case, the Ge Lα line was used for analysis. The accuracy of the measurement of Ge and C was better than 5%.

***Transmittance of the Ge-DLC***: films was measured using a spectrophotometer Shimadzu UV-3600 (Shimadzu, Kyoto, Japan) in a wavelength range from 200 to 3200 nm. A fused silica plate was used as a reference sample, and therefore the transmittance characteristics of the deposited films were measured.

***Cell culture:*** Human hepatocellular carcinoma cell line (Huh7) obtained from the Japanese Collection of Research Bioresources (JCRB) was cultured in Eagle’s minimum essential medium (EMEM; ATCC) supplemented with 10% fetal bovine serum (FBS, Thermo Fisher Scientific) and 0.1% (v/v) penicillin/streptomycin (Sigma, St. Louis, MO) as recommended by the supplier. Cultures were kept in a humidified 5% CO_2_ atmosphere at 37 °C, and the medium was changed once a week [24].

***Cell viability assay***: Cytotoxic activity of the compounds was determined quantitatively by a fluorimetric assay utilizing propidium iodide (PI), as described earlier [25].

Prior to cell seeding, all experimental Ge-based substrates were sterilized by treatment with 70% ethanol for 20 min, followed by ultraviolet (UV) exposure for one hour. For all cell experiments in this study, the cells were seeded on the sterilized substrates at an initial density of 25,000 cells/cm^2^ and were maintained under standard cell culture conditions (37 °C, 5% CO_2_). Cells were allowed to grow on Ge-based substrates for 5 days under standard cell culture conditions [24]. When the cells were in the exponential growth phase, the monolayer was washed once in phosphate-buffered saline (PBS), then stained by the addition of 1 mL PI (50 μg/mL) to each dish for 5 min in the dark at room temperature. The nuclei were counterstained with Hoechst 33,342 (Thermo Fisher Scientific, Waltham, MA, USA). Fluorescence images were recorded with epifluorescent microscope IM-2FL (Optika Microscopes, Ponteranica, Italy). ImageJ (NIH) software was used for image processing and fluorescent micrograph quantification. Cell counting was carried out for five fields of view per sample. Three independent samples per each surface were assessed for cell proliferation measurements, and the reported values are the mean ± SEM (standard error of the mean). PI positive cells were considered to be dead cells. The survival rate was subsequently calculated using the following equation:

Survival rate (%) = (Hoechst positive cells − PI positive cells) × 100/Hoechst positive cells.(1)

As a positive control, the cells were treated with 20% ethanol for 60 min.

Additionally, cell viability was analyzed by alamarBlue assay (Thermo Fisher Scientific, Waltham, MA, USA). AlamarBlue is a resazurin-based solution that functions as a cell health indicator using the reducing power of living cells to quantitatively measure viability. Resazurin, the active ingredient of alamarBlue reagent, is a nontoxic, cell-permeable compound that is blue in color and virtually nonfluorescent. Upon entering living cells, resazurin is reduced to resorufin, a compound that is red in color and highly fluorescent [26]. Cell growth and seeding were the same as for PI measurements. Cells were allowed to grow on Ge-based substrates for 5 days. Afterward, alamarBlue reagent was added to each dish and incubated for 0.5 h at 37 °C to form resorufin. The red fluorescence was measured using a TECAN microplate reader SpectraFluor Plus (TECAN, Mannedorf, Switzerland) at 590 nm. Three independent experiments were performed for each measurement. Cell viability was normalized to control values (no Ge exposure) and expressed as mean ± SEM.

Furthermore, we assessed viability using calcein AM method (Thermo Fisher Scientific, Waltham, MA, USA). Cells were grown for 5 days on germanium substrates as for PI or alamarBlue assays. Calcein AM provides a simple, rapid, and accurate method to measure cell viability and/or cytotoxicity. Calcein AM is a nonfluorescent, hydrophobic compound that easily permeates intact, live cells. The hydrolysis of calcein AM by intracellular esterases produces calcein, a hydrophilic, strongly fluorescent compound that is well retained in the cell cytoplasm [27]. After growing on substrates, cells were stained with calcein-AM (1 μM) for 30 min. The green fluorescence was measured using a TECAN microplate reader SpectraFluor Plus (TECAN, Mannedorf, Switzerland) at 488 nm. Three independent experiments were performed for each measurement. Cell viability was normalized to control values (no Ge exposure) and expressed as mean ± SEM. In order to confirm the validity of the live/dead staining in all cases, cells were also treated with 20% ethanol for 60 min and subsequently analyzed.

***Detection of intracellular reactive oxygen species***: ROS levels were measured using the Cellular ROS/Superoxide Detection Assay Kit (Abcam, Cambridge, UK). ROS levels were assessed as described previously [28]. After growing on Ge substrates, the cells were labeled with fluorescent reporter dyes, which are oxidized by ROS with high specificity, according to the manufacturer’s instruction (Abcam). For total ROS detection, we used the cell permeant reagent 2′,7′-dichlorofluorescein diacetate (DCFDA), a fluorogenic dye that measures hydroxyl, peroxyl, and other ROS activity within the cell. Dihydroethidium (hydroethidine or DHE) was used for superoxide detection. After diffusion in to the cell, DCFDA is deacetylated by cellular esterases to a nonfluorescent compound, which is later oxidized by ROS into 2′,7′-dichlorofluorescin (DCF). DCF is a highly fluorescent compound, which can be detected by fluorescence spectroscopy and/or microscopy with maximum excitation and emission spectra of 495 nm and 529 nm, respectively [29]. DHE has been shown to be oxidized by superoxide to form 2-hydroxyethidium (2-OH-E^+^) (ex 500–530 nm/em 590–620 nm) or by nonspecific oxidation by other sources of ROS to form ethidium (E^+^) (ex 480 nm/em 576 nm) [29]. Thus, one can discriminate specifically the total ROS and superoxide. The nuclei were counterstained with Hoechst 33,342 (Thermo Fisher Scientific, Waltham, MA, USA). Fluorescence images were recorded with epifluorescent microscope IM-2FL (Optika Microscopes, Ponteranica, Italy). ImageJ (NIH) software was used for image processing and fluorescent micrograph quantification. Cell counting was carried out for five fields of view per sample. Three independent samples per each surface were assessed for cell proliferation measurements, and the reported values are the mean ± SEM. Cells treated with H_2_O_2_ (1 mM for 30 min) were used as a ROS positive control.

***Detection of apoptosis***: The Dead Cell Apoptosis Kit (Thermo Fisher Scientific, Waltham, MA, USA) was used to measure early apoptosis by detecting phosphatidylserine expression and membrane permeability. Huh7 were grown on different Ge substrates for 5 days. Afterward, cells were stained with Dead Cell Apoptosis Kit according to the manufacturer’s instructions. Phosphatidylserine expression as an early sign of apoptosis was determined by the binding of Alexa Fluor 488 annexin V. Propidium iodide was used to differentiate necrotic cells. Hoechst 33,342 was used as nucleus staining. After staining, labeled cells were then imaged using spinning disk confocal microscopy IXplore SpinSR10 (Olympus, Tokyo, Japan). ImageJ software (NIH) was used for image processing and quantification. As positive control, 20 µM staurosporine for 2 h was used. ImageJ software was used for image processing and fluorescent micrograph quantification.

***Spinning disk confocal microscopy:*** In order to visualize in great detail the morphological changes of Huh7 upon their growth on Ge substrates, we utilized brand new high-resolution spinning disk confocal microscopy (Spin SR, Olympus). Huh7 were grown on different Ge substrates for 5 days and labeled with propidium iodide—red dye, Hoechst 33,342 nuclear stain—blue. The merging of blue and red gives magenta color. Cell membranes were labeled with CellMask™ Green (green). Labeled cells were then imaged using high-resolution spinning disk confocal microscopy (SpinSR, Olympus). Fluorescence images were taken with the acquisition software cellSens (Olympus). ImageJ software (NIH) was used for image processing.

***Statistical analysis***: Data obtained from independent experiments are presented as the mean ± SEM. Statistical analysis was performed using one-way analysis of variance and the Newman–Keuls test. Differences were considered statistically significant at *p* < 0.05.

## 3. Results and Discussion

### 3.1. Growth Rate, Roughness, Morphology

From the profilometer measurements, we calculated the growth rate. With Ge doping changing from 12 at.% to zero at.%, the growth rate moved from 7.4 × 10^−3^ nm/pulse to 4.0 × 10^−3^ nm/pulse.

**Figure 2:**
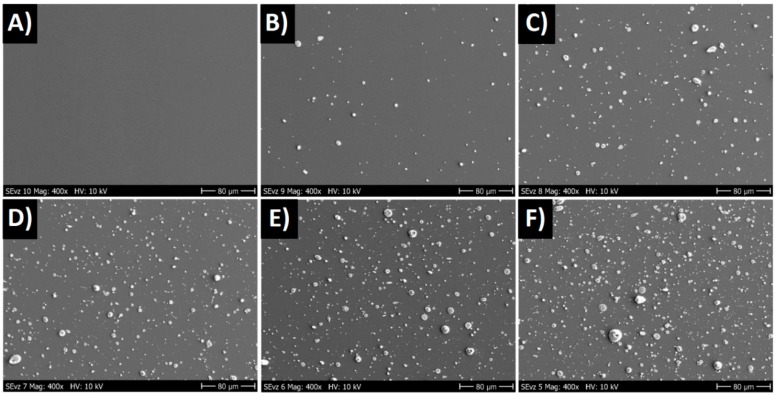
SEM images of Ge-doped DLC layers, 400× magnification. (**A**) 0 at.%, (**B**) 1 at.%, (**C**) 2.5 at.%, (**D**) 5 at.%, (**E**) 9 at.%, and (**F**) 12 at.% of Ge in DLC.

Roughness measurements showed that Ra changed from about 3 nm to 120 nm. From the SEM photos, we could see that the layer consisted of small “grain” droplets, and the surface was covered with grains of sizes up to several micrometers. For lower dopant concentrations, the surface was smoother (see Figure 2).

### 3.2. Composition (WDS)

The measured Ge dopant concentrations moved with deposition conditions from 0.0 at.% to 12 at.% (see Table 1). The WDS analyses showed that the Ge content in the grains was about 10 times higher compared with the smooth parts of the film.

### 3.3. Transmittance

**Figure 3:**
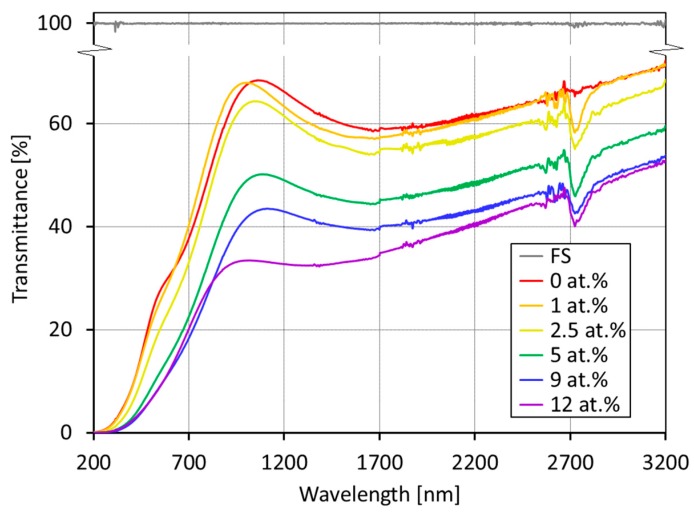
Transmission curves of DLC and Ge-doped DLC films in region up to 3.2 micrometers.

The film transmittance in the UV-VIS and near-infrared regions generally decreased with increasing germanium content (Figure 3). For example, at 1700 nm, the transmittance decreased from 59% for pure DLC (0 at.% of Ge) to 34% for 12 at.% of Ge. The absorption peak around 2700 nm might have been caused by residual OH absorption of fused silica substrates. Our Ge-doped DLC films changed only the value of transparency in UV-VIS but were still transparent compared with pure germanium wafers, which exhibited transparency after ~1.7 micrometers.

### 3.4. In Vitro Biocompatibility Evaluations

Biocompatibility of the materials themselves is a crucial initial step in the research devoted to biomedical applications of any tested material. We first studied how the substrates with different amounts of doped Ge influence the overall growth rate of the cells. Our research was focused on a specific cell type—hepatocytes. These cells are polarized, specialized, and species-specific, making them uniquely susceptible to infections [30]. Therefore, we utilized Huh7 cells for the studies. Huh7 is one of the most widely used in vitro model systems for the study of human hepatocytes [31].

It is worth noting here that for quite some time, germanium and germanium compounds were considered to be safe and used in dietary supplements [32]. Indeed, numerous reports have shown that germanium supplements present a potential hazard to humans at high doses [23]. However, till now, Ge-containing healthcare products are still available [10]. This fact has created a perception that Ge-based implants would be of high biocompatibility and well tolerated by the human body [10,11,12]. Thus, no systemic toxicity of Ge implants in humans has been reported. There is a lack of rigorous analysis of Ge-induced cytotoxicity on relevant cellular models. In fact, detailed studies in the past two decades have shown that redox active metals and metalloids (like Ge) undergo redox cycling reactions and possess the ability to produce reactive oxygen species [23,33,34]. Increased formation of ROS overwhelms cellular antioxidant protection and subsequently induces DNA damage, lipid peroxidation, and cell death [33]. Recently, we showed that among widely used hepatic cell lines, Huh7 are the most susceptible to redox imbalance and oxidative damage and proposed Huh7 a as fragile hepatic cell line [35,36,37]. Thus, to assess acute toxicity of Ge-based substrates, we used Huh7 cell line in our experiments.

**Figure 4:**
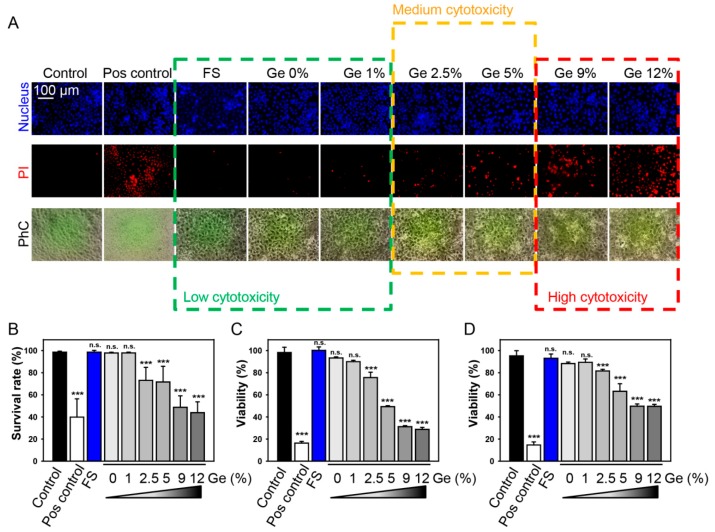
Cell viability of Huh7 cells on different Ge substrates after 5 days of cultivation. (**A**) Cells were stained with propidium iodide (PI, red) to assess cell viability. The nuclei were counterstained with Hoechst blue. Cells were imaged using epifluorescent microscope IM-2FL (OPTIKA Italy). Representative images are shown. As a positive control, cells were treated with 20% ethanol for 60 min. (**B**) Quantitative analysis of survival rate of cells grown on different Ge substrates. ImageJ software (NIH) was used for image processing and quantification. One-way ANOVA with Newman–Keuls multiple comparison test was used. Data are expressed as means ± SEM (*n* = 3), *** *p* < 0.001. (**C**) Cell viability as detected by the alamarBlue assay of Huh7 grown on different Ge substrates for 5 days, *n* = 3 each. The data were normalized to control values (no Ge exposure), which were set as 100% cell viability. As a positive control, cells were treated with 20% ethanol for 60 min. One-way ANOVA with Newman–Keuls multiple comparison test was used. Data are expressed as means ± SEM (*n* = 3), *** *p* < 0.001. (**D**) Cell viability as detected by the calcein AM assay of Huh7 grown on different Ge substrates for 5 days, *n* = 3 each. The data were normalized to control values (no Ge exposure), which were set as 100% cell viability. As a positive control, cells were treated with 20% ethanol for 60 min. One-way ANOVA with Newman–Keuls multiple comparison test was used. Data are expressed as means ± SEM (*n* = 3), *** *p* < 0.001.

Huh7 cells were cultured on substrates with different percentages of doped Ge for 5 days. After 5 days of culturing, the cell viability was assessed utilizing propidium iodide labeling. Propidium iodide (PI) is a well-known membrane impermeant dye that is generally excluded from viable cells. It binds to double-stranded DNA by intercalating between base pairs. Thus, an increase in PI-labeled cells reflects the cytotoxic response. Figure 4A shows the cytotoxic behavior of the cells grown on different Ge substrates as observed by phase-contrast microscopy (PhC) and dead assay by fluorescence imaging. For all samples, most Huh7 cells attached tightly to the surface of the materials and spread effectively during the culture period. Figure 4B summarizes the viability determined by a live/dead cell count from five different areas on each sample. Overall, we were able to subdivide substrates in three categories in accordance with their cytotoxic effects (Figure 4). FS, Ge O%, and Ge 1% substrates had low (nearly no) cytotoxicity; Ge 2.5% and Ge 5% had moderate toxicity; and Ge 9% and Ge 12% had highly toxic (Figure 4). The results gave a substantial indication of the biocompatibility of the investigated substrates. Indeed, propidium iodide staining shows dead cells with already permeabilized membrane. Thus, to assess in more detail the prerequisites of Ge-induced cytotoxicity, we utilized two metabolic activity assays (Figure 4C,D). Consistent with the PI viability results, the viability of Huh7 was concentration-dependently decreased after 5 days of exposure to substrates with different Ge concentrations. Both calcein AM (Figure 4D) and alamarBlue (Figure 4C) assays showed similar results for Ge-induced cytotoxicity. Moreover, viability results from these metabolic activity assays were in line with PI viability results (Figure 4).

**Figure 5:**
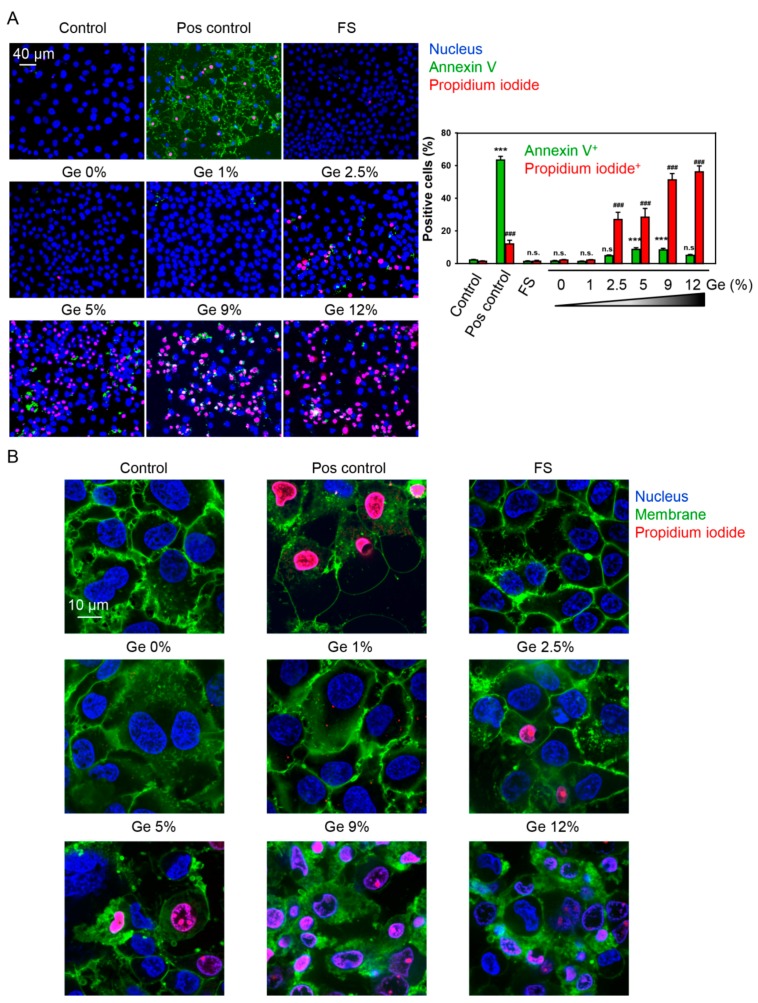
(**A**) Analysis of apoptotic cell death. Huh7 were grown on different Ge substrates for 5 days and labeled with annexin V—green dye, propidium iodide—red dye, Hoechst 33,342 nuclear stain—blue. The merging of blue and red gives magenta color. Labeled cells were then imaged using spinning disk confocal microscopy. ImageJ software (NIH) was used for image processing and quantification. One-way ANOVA with Newman–Keuls multiple comparison test was used. Data are expressed as means ± SEM (*n* = 3), *** *p* < 0.001. As positive control, 20 µM staurosporine for 2 h was used. (**B**) Huh7 were grown on different Ge substrates for 5 days and labeled with propidium iodide—red dye, Hoechst 33,342 nuclear stain—blue. The merging of blue and red gives magenta color. Cell membranes were labeled with CellMask™ Green (green). Labeled cells were then imaged using high-resolution spinning disk confocal microscopy. ImageJ software (NIH) was used for image processing. As a positive control, cells were treated with 20% ethanol for 60 min.

The growth of Huh7 on Ge substrates for 5 days did not induce early signs of apoptosis [38,39], namely, translocation of phosphatidylserine to the outer cell membrane leaflet, as measured by the binding of Alexa488-labeled annexin V with concomitant increase in membrane permeability, as shown by propidium iodide staining (Figure 5A). Indeed, there was minor number of cells positive on annexin V in Huh7 grown on Ge substrates (Figure 5A). In contrast, there was a massive Ge concentration-dependent increase in membrane permeability, as shown by propidium iodide incorporation (Figure 5A). In fact, Huh7 treatment with the well-known compound staurosporine resulted in the formation of distinguished apoptotic hallmarks (Figure 5A). This constellation suggested that Ge substrates resulted in either late stage of apoptotic cell death or some necrotic events.

We further assessed how the morphology of cells changed upon culturing on Ge substrates. We stained cellular membrane with CellMask Green plasma membrane stain. As can be clearly see from Figure 5B and consistent with cell viability and annexin V staining, the cells grown on Ge substrates with 0%–1% germanium showed no significant changes in size, shape, and membrane morphology. However, starting from 2.5% germanium, cells exhibited significant morphological changes (Figure 5B). Huh7 exposure to substrates with high Ge concentrations (≥2.5%) concentration-dependently resulted in massive CellMask intracellular incorporation, indicating membrane permeabilization (Figure 5B). Additionally, culturing on Ge substrates with high Ge concentrations (≥2.5%) resulted in vesicular shedding (Figure 5B). Indeed, multivesicular bodies and microvesicles are shed from the plasma membrane during the cell death process [38]. Such vesicles can be exosomes, apoptotic, or necrotic bodies. To clearly assess the difference, one needs to do a more in-depth study on this matter. In general, vesicular shedding has been implicated in an increasing number of physiological and pathological contexts as mediators of local and systemic intercellular communication [40,41,42]. Such vesicles might augment immune and inflammatory responses [40,41,42]. Importantly, positive control (20% ethanol) treatment showed distinct morphologic changes in comparison with Ge substrates (Figure 5B). No vesicle shedding was observed, and cells showed ballooning morphology, indicating accidental necrotic cell death induced by ethanol (Figure 5B).

The next logical step was to check the ROS accumulation. Excess ROS results in oxidative stress and subsequent cell death. It is well known that ROS are emerging as key effectors in signal transduction [43]. Accumulating evidence also suggests that ROS play a major role in the mediation of metal-induced cellular responses [34]. It is worth noting that the ability of Ge-based materials to produce ROS in cells has not been tested previously [10,11,12].

**Figure 6:**
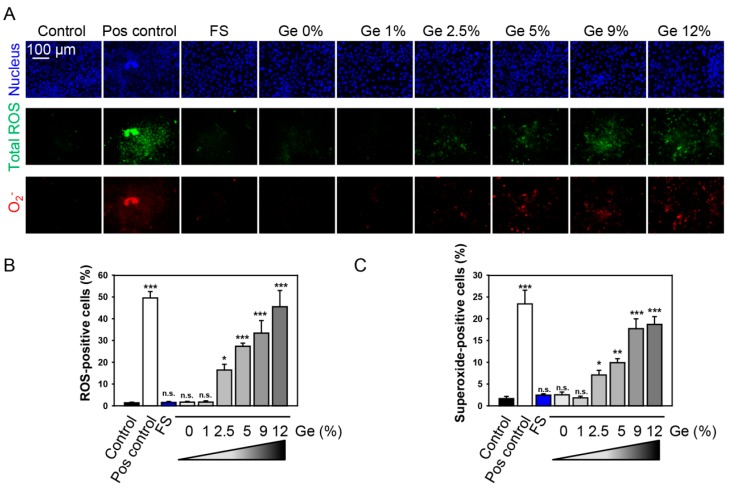
Cell growth on different Ge substrates results in reactive oxygen species (ROS) accumulation. (**A**) Cells were cultured on different Ge substrates for 5 days. Cells were labeled with the ROS-sensitive fluorescent dyes using the cellular ROS/superoxide detection kit (Abcam, Cambridge, United Kingdom). Total ROS were labeled with green dye and superoxide anion with red dye. The nuclei were counterstained with Hoechst blue. Cells were imaged using epifluorescent microscope IM-2FL (OPTIKA Italy). Cells treated with H_2_O_2_ (1 mM for 30 min) were used as a ROS positive control. Representative images out of three independent experiments are shown. (**B**) Quantitative analysis of total ROS-positive cells cultured on different Ge substrates for 5 days. ImageJ software (NIH) was used for image processing and quantification. One-way ANOVA with Newman–Keuls multiple comparison test was used. Data are expressed as means ± SEM (*n* = 3), * *p* < 0.05 ** *p* < 0.01 *** *p* < 0.001. (**C**) Quantitative analysis of total superoxide-positive cells cultured on different Ge substrates for 5 days. ImageJ software (NIH) was used for image processing and quantification. One-way ANOVA with Newman–Keuls multiple comparison test was used. Data are expressed as means ± SEM (*n* = 3), * *p* < 0.05 ** *p* < 0.01 *** *p* < 0.001.

We found that Ge substrates possess a dose-dependent ROS production in Huh7 cells (Figure 6A–C). As can be clearly seen from Figure 6B,C, Ge substrates induced dose-dependent ROS accumulation in cells with the highest amount of ROS, produced after cell culturing on substrates with higher (12%) Ge amount (Figure 6B,C). We used two distinct fluorescent probes. One probe was indicative of cellular production of different ROS types, while the other was superoxide (O_2_^−^)-specific. This allowed us to monitor changes in the total ROS level as well as specifically verify the level of superoxide. Cell culturing on Ge substrates triggered a dose-dependent accumulation of superoxide as well (Figure 6A–C). Indeed, substrates showed dose-dependent ROS and superoxide accumulation, which correlates with cytotoxicity.

**Figure 7:**
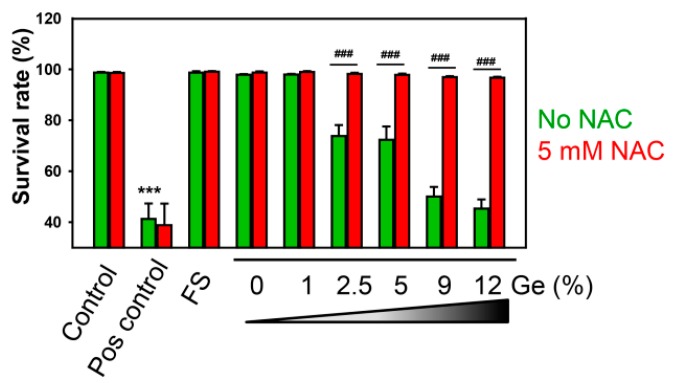
Treatment with ROS scavenging agent *N*-acetyl-L-cysteine (NAC) completely abolished the cytotoxicity of Ge substrates. Huh7 cells were grown on different Ge substrates for 5 days. To inhibit ROS accumulation, Huh7 were supplemented with 5 mM NAC. Cells were stained with propidium iodide to assess cell viability. The nuclei were counterstained with Hoechst blue. Afterward, cells were imaged using epifluorescent microscope IM-2FL (OPTIKA Italy), and quantitative analysis of survival rate of cells was then done using ImageJ software (NIH). One-way ANOVA with Newman–Keuls multiple comparison test was used. Data are expressed as means ± SEM (*n* = 3), *** *p* < 0.001. As a positive control, cells were treated with 20% ethanol for 60 min.

To confirm the role for ROS in the induction of Ge cytotoxicity, we used the ROS scavenger *N*-acetyl-L-cysteine (NAC, a potent ROS scavenger [44,45,46]). As expected, the ROS scavenger was able to antagonize the cytotoxic effects elicited by Ge substrates on Huh7 cells (Figure 7). Importantly, cell death induced by 20% ethanol (used as a positive control) was not antagonized by NAC (Figure 7). High concentrations of ethanol induce accidental necrosis by the direct rupturing of cell membrane without concomitant accumulation of ROS [47]. These data confirmed a pivotal role of ROS accumulation in Ge-induced cytotoxicity.

To summarize this part, we used a hepatic cell line to assess cytotoxicity mediated by Ge substrates and found that concentration of Ge up to 1% in the substrates is not toxic for cell culture. In contrast, concentrations of Ge higher than 5% showed substantial degree of cytotoxicity. Furthermore, we identified the source of Ge-mediated toxicity. Indeed, high Ge concentrations (>2.5%) in the substrates resulted in intracellular ROS production, the accumulation of which leads to cell death.

## 4. Conclusions

Ge-doped DLC layers with Ge doping up to 12 at.% were prepared using two-laser codeposition from Ge and graphite targets. Film properties, such as growth rate, roughness, SEM morphology, WDS composition, VIS-near IR transmittance, and biological properties (cytotoxicity, effects on cellular morphology, and ability to produce ROS) were studied in relation to dopant concentrations. The growth rate of the films was low (from 4.0 × 10^−3^ nm/pulse to 7.4 × 10^−3^ nm/pulse). Roughness (Ra) increased with germanium doping. Transparency decreased with Ge doping, but the shape of the curves was similar to that for pure (undoped) DLC films. Analysis showed that Ge-DLC films exhibit cytotoxicity for higher Ge doping.

Generally, systemic studies on toxicity of Ge are still rather limited [23]. A number of studies have shown that Ge supplements present a potential hazard to humans at high doses [23]. Ge compounds are relatively less toxic compared with other metalloids and metals. However, relatively high doses of germanium dioxide and other inorganic Ge compounds can cause severe poisoning, including death [23]. In the present study, we identified a threshold for Ge concentration in cell culture substrate to avoid severe toxic reaction. We found that Ge concentrations higher than 2.5% induced signs of cell death in hepatic cells. Interestingly, Ge-based materials with Ge concentration as low as 1.5% have already been shown to exhibit cytotoxic effects [10,11,12]. In this regard, our Ge 1% material showed less toxicity in comparison to 1.5 Ge described in [10]. Although ROS produced during physiological processes are rapidly inactivated by antioxidant enzymes, the excess of ROS can induce apoptotic cell death. ROS have been identified as a major reason for metalloids- and metals-induced cytotoxicity [34]. We revealed that Ge concentrations higher than 2.5% resulted in ROS production and cellular accumulation. Excess of ROS production mediated by Ge substrates results in cell death. Therefore, the cytotoxicity of Ge-based materials should be carefully considered when transitioning to clinical practice. Our results imply that the cytotoxic effects of Ge substrates require more intensive study and that they should be considered in biomedical applications.

## Figures and Tables

**Table 1:** Deposition conditions, Ge concentration, and film roughness (Ra) for germanium-doped diamond-like carbon (Ge-DLC) films prepared by double pulsed laser deposition (PLD) arrangement. Film thickness—cca 160 nm. Laser 1—energy density 8 J cm^−2^, spot size 2 × 1 mm^2^. Laser 2—energy density 1.5 J cm^−2^, spot size 4 × 1.5 mm^2^. WDS—wavelength dependent X-ray spectroscopy.

Sample No.	Laser 1—Compex (Graphite Target)		Laser 2—Lumonics (Ge Target)		Ge in DLC (WDS) [at.%]	Roughness Ra [nm]
	No. of Pulses	Rep. Rate [Hz]	No. of Pulses	Rep. Rate [Hz]		
5	21,685	14	29,429	19	12	120.1
6	29,083	30	17,450	18	9	83.6
7	31,891	27	12,992	11	5	63.6
8	36,216	30	6036	5	2.5	61.7
9	38,680	37	2091	2	1	14.4
10	40,000	30	-	-	0	2.8
